# Genetic polymorphisms in *XRCC1* genes and colorectal cancer susceptibility

**DOI:** 10.1186/s12957-015-0650-2

**Published:** 2015-08-15

**Authors:** Yi Huang, Xiaohua Li, Jing He, Lin Chen, Huaxing Huang, Mengdi Liang, Qiannan Zhu, Yaoyu Huang, Li Wang, Chunji Pan, Tiansong Xia

**Affiliations:** The First Affiliated Hospital of Nanjing Medical University, 68 Changle Road, 210006 Nanjing, People’s Republic of China; Emergency Department, Yingtan People’s Hospital of Jiangxi Province, 31 Shenglixi Road, 335000 Yingtan, People’s Republic of China; Breast Center of Jiangsu Province, The First Affiliated Hospital of Nanjing Medical University, 368 Jiangdongbei Road, 210036 Nanjing, People’s Republic of China; The First Clinical Medical College, Nanjing Medical University, 140 Hanzhong Road, 210029 Nanjing, People’s Republic of China

**Keywords:** Colorectal cancer, *XRCC1*, Polymorphism, Molecular epidemiology, Tobacco, Alcohol

## Abstract

**Background:**

The objective of this study is to investigate the association among the polymorphisms of *XRCC1* gene, smoking, drinking, family history of tumors, and the risk of colorectal cancer (CRC) in the population of Han nationality in Jiangsu Province, China.

**Methods:**

A case–control study of 320 patients with CRC and 350 cancer-free subjects as a control group was conducted. The three polymorphic sites, codons 194, 280, and 399, of *XRCC1* genes were analyzed by PCR-RFLP.

**Results:**

We find that heavy smoking (>500 cigarettes per year) significantly increased the susceptibility of CRC (OR = 1.89, 95 % confidence interval (CI) 1.27–2.84) after stratification by total smoking amount. There was also significant difference between cases and controls when family history of tumors (OR = 2.96, 95 % CI 1.76–4.99) was considered. Comparing with individuals carrying XRCC1 399Arg/Arg genotype, the subjects with 399Arg/Gln (OR = 1.46, 95 % CI 1.06–2.01) or 399Gln/Gln genotype (OR = 1.93, 95 % CI 1.05–3.54) had a significantly increased risk for CRC. Taking smoking and drinking habits into consideration, we found that subjects with heavy smoking history and *XRCC1* 194Arg allele had the significantly increased risk for CRC (OR = 2.91, 95 % CI 1.35–6.24). Individuals, who carry 399Gln allele and have a heavy smoking (OR = 2.72, 95 % CI 1.52–4.89) or drinking habit (OR = 1.98, 95 % CI 1.06–3.67), also have higher risk. In smoking population, 194Arg (*P* = 0.491) and 399Gln (*P* = 0.912) had not significantly increased risk for CRC, so did 399Gln (*P* = 0.812) in smoking population.

**Conclusions:**

Individuals carrying *XRCC1* 399Gln allele with a smoking or drinking habit were in increased risk, and heavy-smoking subjects with 194Arg allele also have higher risk for CRC in the Han nationality population of Jiangsu Province, which also showed a positive correlation with exposure dose of tobacco. But *XRCC1* 399Gln allele or 194Arg allele were not independent risk factors for CRC in smoking or drinking population.

## Background

In the Western countries, colorectal cancer (CRC) is the second most common cancer-related cause of death [1]. According to a report of World Health Organization, an estimated 147,500 new cases of colorectal cancer are diagnosed in the world per year, whereas in China, more and more new cases of CRC are diagnosed every year, and now, the risk of CRC is 13.29/100,000 in the Han nationality population of China [2].

Difference in DNA repair capacity (DRC) has been considered to exert an influence on tumor susceptibility [3], and reduced DRC might result in a higher risk for many people in developing cancer [4]. The mechanism of CRC development is similar to other majority tumors, which is dependent on the interactions of genetic factors and environmental agents [2]. Nowadays, the research on the relationship between genotypic polymorphism of DNA repair enzymes and tumor susceptibility has become a focus because living organisms suffer continuous damage from diverse environmental agents and normal cellular metabolism products and DNA repair is essential in protecting the genome of cells from environmental hazards, such as tobacco smoking and alcohol drinking.

Smoking is a rich source of reactive oxygen species and chemical carcinogens, and many studies have proved the close relation between smoking and the development of all kinds of tumors. Reactive oxygen species, coming from burning tobacco, can cause direct damage to DNA by initiating lipid peroxidation and oxidizing proteins. Moreover, chemical carcinogens in cigarette smoke (i.e., polycyclic aromatic hydrocarbons, nitrosamines, and arylamines) can induce bulky adducts in crypt cells, thus also contributing to the formation of mutations in the colon [5]. In addition, alcohol can act as a cocarcinogen by facilitating the absorption of carcinogens, and as a carcinogen due to its conversion to acetaldehyde in the colon lumen, which can form DNA adducts, such as *N*2-ethyl-2-deoxyguanosine and 1,*N*2-propanodeoxyguanosine [6], and induce oxidative DNA damage, such as DNA strand breaks [7].

Although *XRCC1* has no known enzymatic activity, it can interact with several important repair proteins through its different domains, such as DNA ligase III, DNA polymerase β, poly(ADP-ribose) polymerase (PARP) 1 and 2, human AP endonuclease, polynucleotide kinase (PNK), human 8-oxoguanine DNA glycosylase (OGG1), proliferating cell nuclear antigen (PCNA), etc. [8]. A complex system of DNA repair enzymes has a vital role in protecting the genome of cells from all kinds of carcinogenic exposure. The DNA repair enzyme *XRCC1* is thought to be involved in base excision repair (BER) of oxidative DNA and single-strand break (SSB) repair [9].

At present, three common polymorphisms of codon 194, codon 280, and codon 399 [10], which lead to amino acid substitutions in *XRCC1* gene, have been found in the population of Han nationality in China. But, studies focused on the relation between these polymorphic sites, and CRC susceptibility in Han Chinese was few. More importantly, foreign population studies about *XRCC1* polymorphisms related with CRC were inconsistent. Therefore, this study was performed in the Han people of Jiangsu Province in order to reveal the associations among the genetic polymorphisms of *XRCC1* genes, smoking or drinking habit, family history of tumors, and CRC susceptibility.

## Methods

### Study subjects

Between April 2004 and October 2008, a total of 320 CRC patients, who had been diagnosed with CRC through biopsy of fibrocolonoscope or operation, and 350 age- and gender-matched control patients with other nontumorous diseases were included in this study after giving informed consent. The mean age in cases was 64.2 versus 65.3 years in controls. All subjects were ethnic Han Chinese, were able to answer relative questions clearly, and were permanently residing in Jiangsu Province, China. Serological (carcino-embryonic antigen, CEA), physical, and fibrocolonoscope examinations were conducted on all controls to exclude the possibility of CRC, and any control would be excluded from this study if he had any previous cancer diagnosis.

### Data collection

By using a uniform questionnaire, a face-to-face interview, and a review of medical records, we collected some information for every subject, including general characteristics, personal medical history, family cancer history, and history of smoking and alcohol drinking.

Drinking habit was defined as drinking at least three times per week lasting more than 5 years [6, 7]. Family history of tumors was defined as any cancer in first-degree relatives (parents, siblings, or children). In our research, smoking more than five cigarettes per day for more than 5 years was defined as a smoking habit. The unit cigarette × year for evaluating accumulative smoking amount was defined as one cigarette per day for 1 year. Slight smoking was defined when the accumulative smoking amount was no more than (≤) 500 cigarettes per year (the integer of average), and heavy smoking was defined when the accumulative smoking amount was more than (>) 500 cigarettes per year [11, 12].

### Collection for blood specimens

With consent from patients, 5 mL of peripheral vein blood was collected. After anticoagulated with EDTA, the specimens were immediately stored at −70 °C for genotyping.

### Extraction of genome DNA

Genomic DNA was isolated and purified from anticoagulated blood (5 mL) by the traditional phenol/chloroform extraction and ethanol precipitation, dissolved in TE buffer (pH = 7.4), and stored at −20 °C.

#### Analysis of genotypes

Primers were synthesized by Bioasia Co. (Shanghai, China), and 10 polymerase chain reaction (PCR) reaction buffers, 25 mmol/L MgCl2, 10 mmol/L dNTP, Taq DNA polymerase, and four kinds of restriction endonucleases (Pvu II, Rsa I, and Msp I) were all purchased from New England Biolabs (Ipswich, MA, USA).

**Fig. 1:**
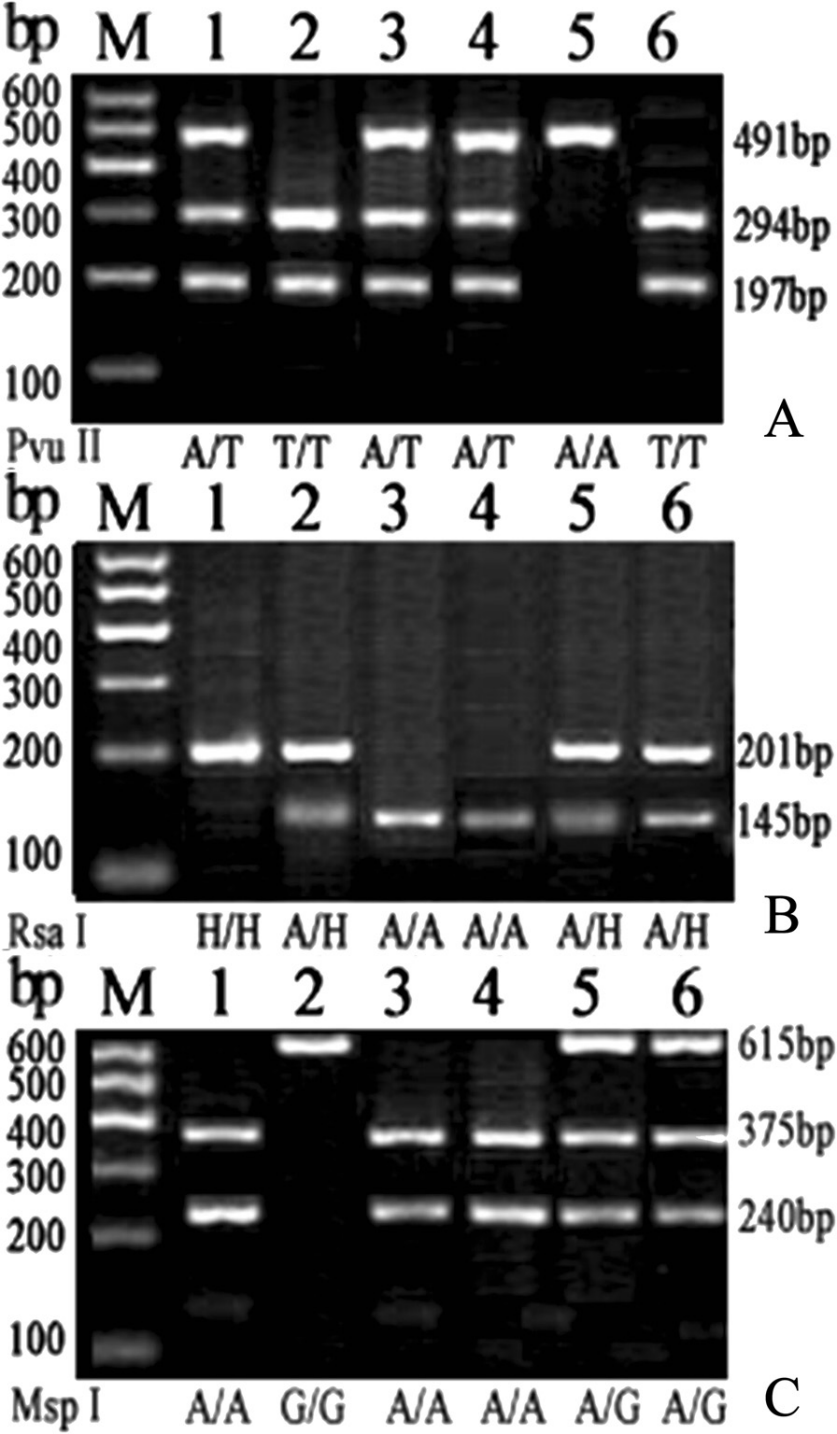
Detection of the genotypes of *XRCC1* Arg194Trp, Arg280His, and Arg399Gln sites. **a** The genotypes of *XRCC1* Arg194Trp sites. **b** The genotypes of *XRCC1* Arg280His sites. **c** The genotypes of *XRCC1* Arg399Gln sites; M: DNA marker ladder; A/A: Arg/Arg; A/T: Arg/Trp; T/T: Trp/Trp; A/H: Arg/His; H/H: His/His; A/G: Arg/Gln; G/G: Gln/Gln; A/T, A/H, A/G: heterozygous genotype

Successfully amplified samples were digested with Pvu II, Rsa I, and Msp I [4, 8, 9, 13]. The volume of enzyme reaction system was 12 μL including 1 μL of 10× buffer, 5 U of restriction endonucleases, 0.1 μL bovine serum albumin, and 10 μL of PCR products. After the reaction system of enzymatic digestion was kept in 37 °C water for 3–6 h, the digested products were allowed to electrophorese on 3 % agarose gels in order to analyze the genotypes of samples (Fig. 1).The *XRCC1* Arg194Trp, Arg280His, and Arg399Gln polymorphisms were determined using the polymerase chain reaction-restriction fragment length polymorphism (PCR-RFLP) method. The primer sequences were selected by referring to published papers and Primer3 software [4, 8, 9, 13]. The total volume of PCR reaction system was 30 μL, containing 3.0 μL of 10 PCR buffer, 2.5 μL of 25 mmol/L MgCl, 0.5 μL of 10 mM/L dNTP, 0.3 μL of 25 μM/L each primer, 0.25 μL of 5 U/mL Taq DNA polymerase, and 100 ng of genomic DNA. The PCR amplification consisted of an initial 5-min incubation at 94 °C, followed by 35 cycles of denaturing at 94 °C for 30 s, various annealing conditions [4, 8, 9, 13], and an extension at 72 °C for 50 s. The reaction was terminated after a final extension of 10 min at 72 °C.

### Statistical analysis

Epidata 3.0 software (EpiData Association, Odense, Denmark) was used to input the data, and SPSS 12.0 software (SPSS, Chicago, IL, USA) was used for statistical analysis. *T* test was used to evaluate mean difference between cases and controls, Hardy–Weinberg test for assessing heredity equilibrium, and *χ*^2^ test for enumeration data and the analysis of their reciprocity. The risk of CRC was evaluated with odds ratio (OR) and 95 % confidence interval (CI).

### Quality control

Investigators were trained uniformly, and in-person interviews were conducted in a hospital. Results of investigation or experiment of 5 % samples were checked randomly. Biolong Gene Limited Company sequenced the PCR amplified products. Data input and process were double tracked, and logic check was adopted.

## Results

### Comparisons of essential features and living habits between cases and controls

There was no significant difference with age group (grouping by 60 years old, the integer of mean age, *P* > 0.05) and gender (*P* > 0.05) between cases and controls. Though we did not find that long-term smoking history increased significantly the risk for CRC (OR = 1.27, 95 % CI 0.93–1.73), heavy smoking (OR = 1.89, 95 % CI 1.27–2.84) significantly increased the susceptibility of CRC after stratification by total smoking amount. There was also significant difference between cases and controls when family history of tumors (OR = 2.96, 95 % CI 1.76–4.99) was considered. No evidence proved that the long-term drinking habit (OR = 1.43, 95 % CI 0.97–2.09) could affect the risk significantly by oneself (Table 1).

**Table 1:** Comparisons of general characteristics and living habits between cases and controls

General characteristic	Control (%)	Case (%)	*χ* ^2^ value	OR	95 % CI
Number of subjects	350	320			
Age group:					
≤60 years	170 (48.6)	152 (47.5)	1	–	–
>60 years	180 (51.4)	168 (52.5)	0.077	1.04	0.77–1.41
Gender					
Female	153 (43.7)	129 (40.3)	1	–	–
Male	197 (56.3)	191 (59.7)	0.794	1.15	0.85–1.56
Family history of tumors:					
No	328 (93.7)	267 (83.4)	1	–	–
*Yes*	*22 (6.30)*	*53 (16.6)*	*17.76*	*2.96*	*1.76–4.99*
Smoking history:					
No	215 (61.4)	178 (55.6)	1	–	–
Yes	135 (38.6)	142 (44.4)	2.322	1.27	0.93–1.73
Total smoking amount:					
Never smoking	215 (61.4)	178 (55.6)	1	–	–
Slight smoking	84 (24.0)	62 (19.4)	0.344	0.89	0.61–1.31
*Heavy smoking*	*51 (14.6)*	*80 (25.0)*	*9.783*	*1.89*	*1.27–2.84*
Alcohol drinking:					
No	290 (82.9)	247 (77.2)	1	–	–
Yes	60 (17.1)	73 (22.8)	3.377	1.43	0.97–2.09

The Italic type meant: this part of data had statistical significance

### Comparisons of genotypic polymorphisms between cases and controls

After analysis by Hardy–Weinberg test, all genotypic frequencies of three polymorphic sites in *XRCC1* gene were ascertained in a balanced state in the Han population of China’s Jiangsu Province according to the heredity-balanced rule.

Comparing with the individuals carrying 399Arg/Arg genotype of *XRCC1* gene, the subjects with 399Arg/Gln (OR = 1.46, 95 % CI 1.06–2.01) or 399Gln/Gln genotype (OR = 1.93, 95 % CI 1.05–3.54) had a significantly increased risk for CRC. However, no other genotypic distribution between cases and controls had significant difference after stratification according to genotype (Table 2).

**Table 2:** Comparison of genotypes of each polymorphic site between cases and controls

Polymorphic site	Genotype	Control (%)	Case (%)	*χ* ^2^ value	OR	95 % CI
Arg194Trp	194Arg/Arg	147 (42.0)	151 (47.2)	1	–	–
(C26304T)	194Arg/Trp	167 (47.7)	141 (44.1)	1.452	0.82	0.60–1.13
	194Trp/Trp	36 (10.3)	28 (8.75)	1.010	0.76	0.44–1.30
Arg280His	280Arg/Arg	252 (72.0)	218 (68.1)	1	–	–
(G27466A)	280Arg/His	86 (24.6)	94 (29.4)	1.778	1.26	0.90–1.78
	280His/His	12 (3.43)	8 (2.50)	0.315	0.77	0.31–1.92
Arg399Gln	399Arg/Arg	205 (58.6)	154 (48.1)	1	–	–
(G28152A)	*399Arg/Gln*	*125 (35.7)*	*137 (42.8)*	*5.367*	*1.46*	*1.06–2.01*
	*399Gln/Gln*	*20 (5.70)*	*29 (9.06)*	*4.624*	*1.93*	*1.05–3.54*

The Italic type meant: this part of data had statistical significance

### Interaction between living habits and genetic polymorphisms in CRC risk

According to the results listed in Table 2, we set individuals with *XRCC1* 194Trp/Trp, 280Arg/Arg, or 399Arg/Arg genotype as the baseline for statistical analysis of interaction between living habits and genetic polymorphisms.

In addition, the subjects who carried *XRCC1* 399Gln allele and had a drinking habit were in a significantly higher risk for CRC in comparison with the subjects who carried 399Arg/Arg genotype and had no drinking habit (OR–1.98, 95 % CI 1.06–3.67). But, we did not find any interaction between the other two polymorphic sites (Arg194Trp and Arg280His) of *XRCC1* gene and drinking habit, which significantly affected the susceptibility of CRC (Table 3).

**Table 3:** Influence of interaction between living habits and genetic polymorphisms on the risk of CRC

Factor 1	Factor 2	Controls (%)	Cases (%)	*χ* ^2^ value	OR	95 % CI
Total smoking amount	Arg194Trp					
Never smoking	Trp/Trp	25 (7.14)	13 (4.06)	1	–	–
Slight smoking	Arg/Trp + Arg/Arg	77 (22.0)	56 (17.5)	0.765	1.40	0.66–2.97
*Heavy smoking*	*Arg/Trp + Arg/Arg*	*47 (13.4)*	*71 (22.2)*	*7.794*	*2.91*	*1.35–6.24*
Total smoking amount	Arg280His					
Never smoking	Arg/Arg	160 (45.7)	116 (36.3)	1	–	–
Slight smoking	Arg/His + His/His	28 (8.00)	22 (6.88)	0.067	1.08	0.59–1.99
Heavy smoking	Arg/His + His/His	15 (4.29)	18 (5.63)	1.880	1.66	0.80–3.42
Total smoking amount	Arg399Gln					
Never smoking	Arg/Arg	130 (37.1)	81 (25.3)	1	–	–
Slight smoking	Arg/Gln + Gln/Gln	37 (10.6)	30 (9.38)	0.865	1.30	0.75–2.27
*Heavy smoking*	*Arg/Gln + Gln/Gln*	*23 (6.57)*	*39 (12.2)*	*11.69*	*2.72*	*1.52–4.89*
Drinking habit	Arg194Trp					
No	Trp/Trp	32 (9.14)	19 (5.94)	1	–	–
Yes	Arg/Trp + Arg/Arg	56 (16.0)	64 (20.0)	3.704	1.93	0.98–3.77
Drinking habit	Arg280His					
No	Arg/Arg	213 (60.9)	173 (54.1)	1	–	–
Yes	Arg/His + His/His	21 (6.00)	28 (8.75)	2.657	1.64	0.90–2.99
Drinking habit	Arg399Gln					
No	Arg/Arg	166 (47.4)	108 (33.8)	1	–	–
*Yes*	*Arg/Gln + Gln/Gln*	*21 (6.00)*	*27 (8.44)*	*4.754*	*1.98*	*1.06–3.67*

The Italic type meant: this part of data had statistical significance

After stratification by accumulative smoking amount of 500 cigarettes per year, the risk for CRC increased significantly in the subjects with heavy smoking history (>500 cigarettes per year) and *XRCC1* 194Arg allele (194 Arg/Trp or 194Arg/Arg) in comparison with the subjects who were 194Trp/Trp genotype and never smoked (OR = 2.91, 95 % CI 1.35–6.24). Meanwhile, we also found that the individuals with heavy smoking history and 399Gln allele (399Arg/Gln or 399Gln/Gln) had significantly increased risk when compared with individuals who carried 399Arg/Arg genotype and never smoked (OR = 2.72, 95 % CI 1.52–4.89). There was no significant interaction between slight smoking history (≤500 cigarettes per year) and genetic polymorphisms of *XRCC1* gene, and we also did not discover that people with heavy smoking history and 280His allele (280 Arg/His or 280His/His) had higher risk than people who had no smoking habit and with 280 Arg/Arg genotype (OR = 1.66, 95 % CI 0.80–3.42) (Table 3). Then, we calculated the risk of three significant groups in Table 3 in smoking or drinking population individually. It showed no significantly increased risk related to *XRCC1* 399Gln allele or 194Arg allele in drinking people or 194Arg allele in smoking people (Table 4).

**Table 4:** Influence of genetic polymorphisms on the risk of CRC in smoking or drinking people

	Controls (%) *n* = 350	Cases (%) *n* = 320	*P*
Arg194Trp (smoking)			
Trp/Trp	11(3.1)	15(4.7)	0.491
Arg/Trp + Arg/Arg	124(35.4)	127(39.7)	
Arg399Gln (smoking)			
Arg/Arg	122(34.9)	137(42.8)	0.912
Arg/Gln + Gln/Gln	60(17.1)	69(21.6)	
Arg399Gln (drinking)			
Arg/Arg	39(11.1)	46(14.4)	0.812
Arg/Gln + Gln/Gln	21(6.0)	27(8.4)	

## Discussion

According to the epidemiological analysis of questionnaire data, we found that family history of tumors significantly increased the susceptibility of CRC, which showed the hereditary basis of patients with CRC, just like mostly tumorous patients. Although we did not find that only smoking habit increased significantly the risk of CRC, after stratification by total smoking amount, heavy smoking history significantly increased the susceptibility of CRC. It seemed that there lay a dosage–response relation during the process of cigarette carcinogenesis by the increased intake of reactive oxygen species and chemical carcinogens, such as olycyclic aromatic hydrocarbons, nitrosamines, and arylamines [5].

Abdel-Rahman et al. reported that *XRCC1* 399Gln allele might be associated with the increased risk for CRC in Egypt (OR = 3.98, 95 % CI 1.50–10.6) [14]. In Korea, Hong et al. also discovered higher risk of 399Gln allele (OR = 1.61, 95 % CI 1.09–2.39) [15]. Our results showed that the subjects with 399Arg/Trp (OR = 1.46, 95 % CI 1.06–2.01) or 399Trp/Trp genotype (OR = 1.93, 95 % CI 1.05–3.54) had a significantly increased risk for CRC, when compared with the individuals carrying 399Arg/Arg genotype of *XRCC1* gene, which was consistent with the above-mentioned findings in Egypt and Korea. But, these findings were not proved in the studies conducted in a Norwegian population; Skjelbred et al. found that the *XRCC1* 399Gln allele was significantly associated with a reduction of CRC risk (OR = 0.62, 95 % CI 0.41–0.96) [16]. It suggests that *XRCC1* polymorphisms may play different roles in different populations and the precise mechanism needs further studies with more samples.

Human *XRCC1* gene is mapped at human chromosome 19q13.2–13.3, spans a genomic distance 33 kb, contains 17 exons, and transcripts a protein of 633 amino acids (69.5 kDa) [17]. In this study, we investigated the associations of three polymorphisms of the DNA repair gene *XRCC1* with the risk of CRC in a southern Chinese population. The Arg399Gln polymorphism is located in the region of the breast cancer susceptibility gene C terminus I (BRCT-I) interaction domain of *XRCC1* with poly(ADP-ribose) polymerase, and the presence of the variant 399Gln allele has been shown to be associated with measurable reduced DRC, as assessed by the persistence of DNA adducts [18, 19], tumor-suppressor gene P53 mutations [20], increased red blood cell glycophorin A [18], elevated levels of sister chromatid exchanges [19], and prolonged cell-cycle delay [21].

Both the *XRCC1* Arg194Trp and Arg280His variants occur in the newly identified PCNA binding region [22]. The *XRCC1* Arg280His variant protein has an ineffective or reduced ability to localize a damaged site in the chromosome, thereby reducing the cellular BER/SSB repair efficiency. The variant 280His allele may allow un-repaired SSBs to accumulate, thereby accelerating genomic instability which consequently increases the risk of carcinogenesis [23]. Only a few studies have investigated the association between the *XRCC1* 280His allele and risk of cancer. Skjelbred et al. in Norwegian found that 280His allele was significantly associated with an increased risk of colorectal adenomas (OR = 2.30, 95 % CI 1.19–4.46), but among the carcinoma cases, the same result was not observed (OR = 1.48, 95 % CI 0.29–7.48) [16]. Also, no significant association has been observed with CRC in the study conducted by Hong et al. in a Korean population [15], and we also did not find significant association between the *XRCC1* 280His allele and colorectal carcinoma risk, which showed consistency with the result of Hong et al. in Asian people.

Few studies have examined the influence of the 194Trp allele on the function of the *XRCC1* protein, but several researches have demonstrated same results in different populations. The study conducted by Abdel-Rahman et al. in Egypt showed that people with 194Trp allele did not tend to be in higher risk for CRC (OR = 2.56, 95 % CI 0.73–9.40) [14]. Hong et al. in Korean [15] and Skjelbred et al. in Norwegian [16] also reported that *XRCC1* 194Trp allele did not significantly increase CRC risk. Our finding was also in accordance with the above-mentioned reports and showed that 194Arg/Trp and 194Trp/Trp genotypes both did not increase the risk for CRC when comparing with 194Arg/Arg genotype.

The *XRCC1* protein plays a key role in base excision repair where it coordinates all the steps by serving as a scaffold via its interaction with other key base excision repair proteins, such as DNA polymerase β, DNA ligase III, polynucleotide kinase 3′-phosphatase, poly(ADP-ribose) polymerase, APE1, and OGG1. We analyzed the interaction of genetic polymorphisms of *XRCC1* gene and unhealthy life habits, such as smoking and drinking habits, because many studies suggested that there lay widespread synergisms. A recent review of the literature found an overall modification of the effect of smoking by *XRCC1* across several cancer types [24, 25]. So, the activity of *XRCC1* protein is crucial to individuals to prevent the formation of mutations induced by chemical carcinogens in cigarette smoke. But, very few researches have discussed the interaction between smoking habit and *XRCC1* genetic polymorphisms in CRC risk. In our results, there was no significant interaction between slight smoking history and all three genetic polymorphisms of *XRCC1* gene after stratification by accumulative smoking amount, but we found that the individuals with heavy smoking history and 399Gln or 194Arg allele had significantly increased risk when compared with individuals who never smoked and carried 399Arg/Arg or 194Trp/Trp genotype, which seemed to be inconsistent with the finding observed by Stern et al. in Singapore Chinese. And, we did not do the associated analysis of haplotype and smoking habit because of the insufficient sample size. Besides, when we took *XRCC1* 399Gln allele or 194Arg allele into analysis for CRC in smoking or drinking population, it did not show significant relevance. This might also be because of the insufficient sample size.

Stern et al. in an American population found that the association between ever smoking and colorectal adenoma risk was only observed among subjects who carried the *XRCC1* codon 194 Arg/Arg and codon 399 Arg/Arg or 399Arg/Gln genotypes (test of interaction, *P* = 0.048) [4]. In Singapore Chinese, Stern et al. observed evidence that *XRCC1* might modify the effects of smoking (interaction *P* = 0.012). The effect of smoking among carriers of the Arg(194)-Gln(399) haplotype was OR = 0.7 (95 % CI 0.4–1.1), whereas, among carriers of the Trp(194)-Arg(399) haplotype, it was OR = 1.6 (95 % CI 1.1–2.5) [3].

In addition, the interaction between three polymorphic sites of *XRCC1* gene and drinking habit was also evaluated in this study, and it only showed a higher risk in subjects with 399Gln allele and drinking habit than individuals carrying 399Arg/Arg genotype but never drinking. Stern et al. in Singapore Chinese also observed a positive association between *XRCC1* codon 194Trp/Trp genotype and alcohol consumption (OR = 2.8, 95 % CI 1.0–8.1), and thought a role for reactive oxygen species as relevant genotoxins that might account for the effects of both smoking and alcohol on colorectal cancer risk [3]. Hong et al. found that alcohol consumption (≥80 g/week) is a significant risk factor of CRC (OR = 2.60, 95 % CI 1.46–4.62), but they did not analyze the interaction of *XRCC1* polymorphisms and drinking habit [15]. So, we considered that the decreased *XRCC1* protein activity in response to chronic alcohol consumption probably finally contributed to the higher risk of CRC, though the mechanism was still not clear.

## Conclusions

Taken together, the individuals, who are with *XRCC1* 194Arg allele and heavy smoking history, or who carry *XRCC1* 399Arg/Gln or 399Gln/Gln genotype and have a heavy smoking or drinking habit, have the increased risk for CRC in the population of Han nationality in Jiangsu Province of China which also shows a positive correlation with exposure dose of tobacco, but these *XRCC1* polymorphisms could not be independent risk factors in smoking or drinking population. The etiology of CRC was unlikely to be explained only by genetic polymorphisms because hereditary variation, itself, could not affect the risk of any disease. Future molecular epidemiology studies should take more environmental risk factors into consideration for CRC.
